# The uncertain correlation of ANCAs in patients with lupus nephritis and crescents, an experience from Chinese centers

**DOI:** 10.1007/s40620-024-02085-5

**Published:** 2024-10-10

**Authors:** Qiong Zhang, Fei Zhou, Yan Zhao, Congjuan Luo, Yankun Luo, Yun Zhou

**Affiliations:** 1 Department of Nephrology, Fifth Hospital of Shanxi Medical University (Shanxi Provincial People’s Hospital), Taiyuan, China; 2 Shanxi Genetic Engineering Center for Experimental Animal Models, Taiyuan, China; 3 Shanxi Provincial Key Laboratory of Kidney Disease, Shanxi Provincial People’s Hospital, Taiyuan, China; 4 Department of Nephrology, Zibo Municipal Hospital (Binzhou Medical College Affiliated Hospital), Zibo, China; 5 Department of Nephrology, The Affiliated Hospital of Qingdao University, Qingdao, Shandong China; 6 Department of Nephrology, Shanxi Province Integrated Traditional and Western Medicine Hospital, Taiyuan, China

**Keywords:** Anti-neutrophil, Systemic lupus erythematosus, Crescentic lupus nephritis

## Abstract

**Background:**

The precise role of anti-neutrophil cytoplasmic antibodies (ANCAs) in the pathologic course of crescentic lupus nephritis (LN) remains unclear. Our study aimed to assess whether ANCA-positive serology in patients with LN and crescents is associated with different clinicopathologic features and outcomes.

**Methods:**

We reviewed the records of 658 patients diagnosed with LN between 2010 and 2022. Among them, 64 (9.7%) patients who had complete follow-up and clinical data were reclassified as crescentic glomerulonephritis. Of these, 11 patients with incomplete ANCA data and 7 patients with less than 10 glomeruli under light microscopy were excluded; ultimately, 46 patients were enrolled: 12 with ANCA positivity and 34 with ANCA negativity. Clinicopathological characteristics and outcomes were analysed and compared.

**Results:**

Our data did not reveal any differences in clinical or laboratory parameters or histopathological features except for a significantly higher level of proteinuria or proportion of nephrotic syndrome (*p* < 0.05) at presentation before biopsy in the ANCA-negative group than in the ANCA-positive group,and a lower level of serum albumin (*p* < 0.05) in the ANCA-negative group than in the ANCA-positive group. No significant differences in complete remission or partial response were detected between the two groups based on the 2021 KDIGO criterion.

**Conclusion:**

Short-term follow-up (average follow-up time of less than 3 years) did not reveal any difference in outcomes between ANCA-positive and ANCA-negative crescentic LN. However, the role of ANCAs in the pathological course of crescentic lupus nephropathy and the effect of ANCAs on long-term outcomes remain to be determined.

**Graphical abstract:**

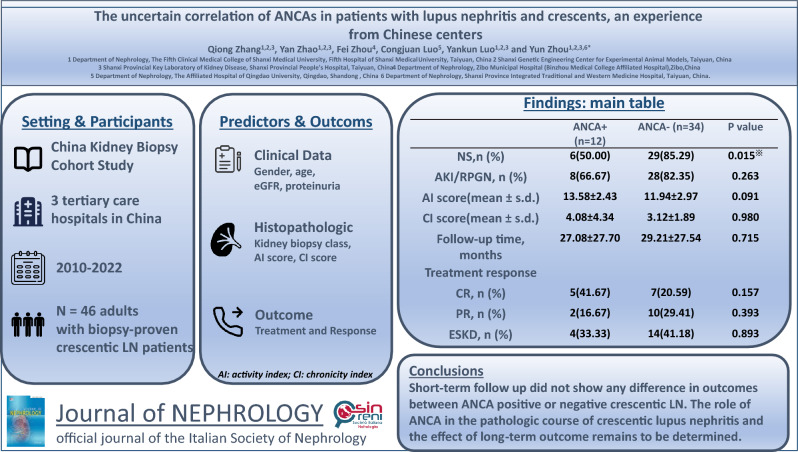

## Introduction

Systemic lupus erythematosus (SLE) is a disease involving multiple organs and is associated with several serum autoantibodies, including anti-neutrophil cytoplasmic antibodies (ANCAs). The reported prevalence of ANCAs varies greatly, with some studies showing a prevalence ranging from none to 30% [1, 2]. Despite the relatively high prevalence of these conditions, it remains unclear whether they are related to any particular clinical feature of lupus. Some studies have reported an association between this histopathologic phenotype and ANCA positivity, but others have found no association [3, 4]. The extensive necrotic, pauci-immune glomerular inflammation and crescents observed in some patients with Class IV lupus nephritis (LN) may be associated with ANCAs [5, 6]. Several studies have reported that crescentic LN, defined as greater than or equal to 50% crescents, is a risk factor for kidney failure and death.

In some studies, LN and ANCA positivity were associated with a severe clinical course [6]. Treatment for LN and ANCA-associated vasculitis usually requires a more intensive immunosuppressive therapy to improve short-term survival, but high rates of infection may affect long-term survival [7]. The precise role of ANCAs in the pathologic course of crescentic LN has yet to be determined. The aim of our study was to estimate whether there is a relationship between ANCA positivity, the clinicopathological features of crescentic LN and kidney and patient outcomes.

## Materials and methods

### Patients

This retrospective case‒controlled study was conducted between 2010 and 2022 at the Department of Nephrology, Shanxi Provincial People’s Hospital, Taiyuan, Zibo Municipal Hospital, Shandong Province and the Affiliated Hospital of Qingdao University, Qingdao. During the study period, a total of 658 Chinese patients with biopsy-proven LN were enrolled in the study. Patients who fulfilled the following criteria were included in this study: [i] consistent with LN type IV and number of crescents greater than 50% of the glomeruli based on the revised International Society of Nephrology/Renal Pathology Society (ISN/RPS) 2018 classification [8]; [ii] tested for ANCA specificity; [iii] duration of follow-up ≥ 6 months; [iv] patients with ≥ 10 glomeruli under light microscopy; and [v] complete baseline and follow-up data. Simultaneously, ANCA-negative crescentic LN patients were included as the control group and were compared with ANCA-positive crescentic LN patients.

### Renal morphology

For light microscopy, we processed biopsy specimens for Hematoxylin & Eosin (H&E), periodic acid-Schiff (PAS), Masson trichrome and silver methenamine staining. Pathological parameters such as activity index and chronicity index were determined via modification of a previously reported system involving the semi-quantitative scoring of specific biopsy features. Biopsy specimens were reviewed and reclassified according to the revised ISN/RPS 2018 classification. We determined the fraction of glomeruli with cellular or fibrocellular crescents based on the 2018 ISN/RPS revised LN classification system. A crescent was defined as a lesion consisting of extracapillary hypercellularity and composed of a variable mixture of cells. Fibrin and fibrous matrix may be present in 10% or more of the circumference of Bowman’s capsule. A cellular crescent was defined as more than 75% cells and fibrin and less than 25% fibrous matrix, and a fibrocellular crescent was defined as 25–75% cells and fibrin and the remainder fibrous matrix. Two renal pathologists examined each biopsy specimen.

### Data collection

The following data were collected retrospectively at biopsy: sex, age, body mass index (BMI), duration of LN, SLE disease activity index (SLEDAI), 24-h urinary protein excretion, serum albumin, serum creatinine (Scr), estimated glomerular filtration rate (eGFR), C3 and C4 levels, antinuclear antibody (ANA), anti-dsDNA, activity index score, and chronicity index score.

Patients who were given methylprednisolone pulse therapy received one to three injections of 250 mg–500 mg/day for 3 days followed by oral corticosteroids at doses of 0.6–0.8 mg/kg body weight/day. Patients who were given cyclophosphamide received a dose of 0.5–0.75 g/m^2^ body surface area once a month for 6 months, while keeping the total dose of cyclophosphamide to less than 9 g. In patients who were given mycophenolate mofetil, the prescribed doses were 1–2 g/day for 6 months. Maintenance therapy was started at the end of induction therapy, with lower doses of prednisone and immunosuppressants. At the end of the follow-up period, we recorded the patients’ laboratory findings at the last visit.

### Detection of ANCAs in sera of LN patients

ANCA was assessed by both an indirect immunofluorescence assay and an antigen-specific enzyme-linked immunosorbent assay (ELISA). A standard indirect immunofluorescence assay was carried out using pre-cooled ethanol-fixed normal peripheral neutrophils as a substrate according to the manufacturer’s instructions (Euroimmun, Lubeck, Germany). The use of Hep-2 cells and paraformaldehyde-fixed neutrophils allowed us to distinguish between anti-nuclear antibodies and p-ANCA. In antigen-specific ELISA, highly purified human neutrophil myeloperoxidase (MPO), purified as reported earlier, was used as a solid-phase ligand to detect MPO–ANCA, and an earlier described sandwich ELISA was used to detect PR3-ANCA.

### Assessments and endpoints

The included outcome measures were retrospectively analysed.

LN remission included complete remission and partial remission, defined based on the KDIGO 2021 Clinical Guideline for the Management of Glomerular Diseases [9]. Complete remission was defined as proteinuria < 0.5 g/day based on a 24-h urine sample with stabilisation or improvement in kidney function (± 10–15% of baseline) within 6–12 months of starting therapy. For children < 18 years old, complete response was defined as proteinuria < 0.5 g/1.73 m^2^/d or < 300 mg/m^2^/d based on a 24-h urine specimen. Partial response was defined as a reduction in proteinuria of at least 50% to < 3 g/day with stabilisation or improvement in kidney function (± 10%–15% of baseline) within 6–12 months of starting therapy, while failure to achieve a partial or complete response within 6–12 months of starting therapy was defined as no response.

A relapse was defined as renal relapse, proteinuria ≥ 1.0 g/24 h in patients with complete response or proteinuria ≥ 2.0 g/24 h in patients with partial response, with or without active urine sediment or an increase in Scr of ≥ 30%. The patients were followed up in an outpatient clinic specific for patients with LN.

A combined event of end-stage kidney disease ([ESKD]; eGFR < 15 mL/min/1.73 m^2^, requirement for maintenance dialysis for at least 6 months, or kidney transplantation) or death was recorded during follow-up.

Informed consent was obtained from each patient for renal biopsy. The research complied with the Declaration of Helsinki.

### Statistical analyses

The data were analysed using SPSS version 26.0 (Statistical SPSS, Inc., Chicago, IL, USA). Data are expressed as mean ± standard deviation (SD) or median range, as appropriate. *p* values less than 0.05 were considered to indicate statistical significance in all analyses. All parameters were compared using a *χ*^2^ test or Fisher’s test for categorical data, and a *t*-test and the Mann‒Whitney *U* test were used for continuous data.

## Results

### Patient information

Among the 658 patients with LN, 64 (9.7%) were classified as with crescentic glomerulonephritis. Of these, 11 patients with incomplete ANCA detection and missing immunofluorescence assay or an antigen-specific ELISA [2], and 7 patients with fewer than 10 glomeruli under light microscopy were excluded; ultimately, 46 patients were selected. Nine patients were male, 37 were female, the average age was 33.98 ± 14.43 (14 to 67) years at presentation, and 36 (78.26%) patients presented with clinical rapidly progressive glomerulonephritis (RPGN) or AKI. The median serum creatinine level was 216.23 (122.15, 438.30) µmol/l at diagnosis, and the median proteinuria level was 5.33 (3.38, 7.78) g/d at diagnosis. The median duration of LN was 18.02 (0.00, 54.00) months. In total, 36/46 (78.3%) patients with crescentic LN presented with acute kidney injury and rapidly progressive glomerulonephritis. On renal pathological evaluation, the median percentage of glomeruli with crescents was 68% (57%, 82.3%) (range 50–96%), and 18/46 (39.1%) patients developed ESKD during follow-up.

Twelve (25.5%) patients showed anti-ANCA positivity by indirect immunofluorescence assay, including 7 with p-ANCA positivity, 4 with MPO-ANCA positivity, 1 with PR3-ANCA positivity and 34 with ANCA negativity by ELISA.

### Clinical and laboratory parameters

The clinical features and pathological findings of ANCA positive and ANCA negative patients are listed in Table 1. There were no significant differences in demographic features (sex, age or BMI) or the time between the presentation of LN before biopsy or treatment between the two groups. There were no significant differences in the incidence of hypertension, serum creatinine level, C3 level, SLEDAI score, or the serum anti-ANA concentration between the two groups. The observed extrarenal manifestations are summarised in the table. We did not observe any differences when comparing crescentic LN patients with and without ANCA positivity.

**Table 1:** Clinical features and pathological findings based on ANCA specificity

	ANCA + (*n* = 12)	ANCA– (*n* = 34)	*p* value
Male/female	3/12	6/34	0.898
Age (years)	32.00 (22.8, 59.5)	29.00 (24.0, 34.5)	0.391
BMI	23.84 ± 3.20	24.21 ± 4.13	0.97
Duration of LN, months	0.00 (0.0, 60.0)	36.00 (0.0, 54.0)	0.331
SLEDAI, score	19.25 ± 5.15	16.94 ± 5.07	0.08
Malar rash, *n* (%)	2 (16.67)	1313 (38.24)	0.311
Photosensitivity, *n* (%)	11 (91.67)	31 (91.18)	0.586
Oral ulcer, *n* (%)	0 (0.00)	5 (14.71)	0.386
Alopecia, *n* (%)	5 (41.67)	8 (23.53)	0.408
Arthralgia, *n* (%)	3 (25.00)	14 (41.18)	0.516
Serositis, *n* (%)	7 (20.59)	4 (33.33)	0.620
Neurological disorder, *n* (%)	0 (0.00)	1 (2.94)	0.947
Hypertension, *n* (%)	8 (66.67)	26 (76.47)	0.777
Proteinuria, g/24 h	3.57 (2.2, 5.3)	6.81 (4.1, 9.8)	0.004^※^
Haematuria, *n* (%)	11/12	31/34	0.586
Albumin, g/L	29.01 ± 4.99	24.61 ± 5.42	0.018^※^
Scr, umol/L	241.43 (83.1, 398.6)	204.61 (140.8, 473.4)	0.412
NS, *n* (%)	6 (50.00)	29 (85.29)	0.015^※^
AKI/RPGN, *n* (%)	8 (66.67)	28 (82.35)	0.263
C3(g/L)	0.43 ± 0.12	0.46 ± 0.13	0.435
Positive ANA, *n* (%)	11 (91.67)	32 (94.12)	0.77
Positive anti-ds-DNA, *n* (%)	7 (58.33)	20 (58.82)	0.977
Thrombocytopenia, *n* (%)	1 (8.33)	4 (11.76)	0.833

*ANCA* Antineutrophil cytoplasmic antibody; *LN* lupus nephritis; *SLEDAI* systemic lupus erythematosus disease activity index; *ALB* serum albumin; *Scr* serum creatinine; *AKI* acute kidney injury; *RPGN* rapidly progressive glomerulonephritis. *C3* serum C3; *ANA* antinuclear antibodies, *ds-DNA* double-stranded deoxyribonucleic acid

※*p* < 0.05 when comared with ANCA – group

We observed a significantly lower level of proteinuria, a lower proportion of nephrotic syndrome (*p* < 0.05) and a higher level of serum albumin (*p* < 0.05) in the ANCA-positive group than in the ANCA-negative group.

### Histopathological features

Renal biopsy findings are shown in Table 2. Among the 12 ANCA-positive LN patients who displayed crescents in the kidney biopsy, 9 (75%) presented with class IV, and 3 (25%) presented with class IV + V. Among the 34 ANCA-negative patients, 26 (76.5%) presented with class IV, and 8 (23.5%) with class IV + V.

**Table 2:** Pathological findings based on ANCA specificity

	ANCA + (*n* = 12)	ANCA– (*n* = 34)	*p* value
Light microscopy			
Kidney biopsy class			
IV, *n* (%)	9 (75%)	26 (76.5%)	0.918
IV + V, *n* (%)	3 (25%)	8 (23.5%)	0.918
Number of glomeruli	36.5 (27, 44.8)	27 (18, 38.3)	0.052
AI score	14.00 (12.3, 15.0)	13.00 (10.0, 14.0)	0.087
Total crescents	24.5 (19.8, 27.8)	19.5 (12.8, 25)	0.04^※^
Total crescents/number of glomeruli	0.735 (0.6, 0.9)	0.665 (0.6, 0.8)	0.499
Cellular crescents	9.0 (5.3, 15.5)	6.5 (2.8, 11.3)	0.275
Cellular crescents/number of glomeruli	0.31 (0.1, 0.6)	0.265 (0.1, 0.5)	0.592
Endocapillary hypercellularity	3.00 (2.3, 3.0)	3.00 (2.0, 3.0)	0.663
Fibrinoid necrosis	0.00 (0.0, 1.8)	0.00 (0.0, 1.0)	0.712
Subendothelial hyaline deposits	1.00 (0.0, 2.0)	1.00 (0.0, 2.0)	0.611
Glomerular leukocyte infiltration	3.0 (1.0, 3.0)	1.5 (1.0, 3.0)	0.062
Interstitial inflammation	1.00 (1.0, 2.0)	0.00 (0.0, 2.0)	0.154
Fibro-cellular crescents	6.00 (3.0, 13.5)	6.00 (3.0, 12.5)	0.598
CI score	2.50 (2.0, 4.8)	3.00 (2.0, 4.0)	0.844
Fibrous crescents	0.00 (0.0, 2.8)	0.00 (0.0, 0.5)	0.612
Fibrous crescents/number of glomeruli	0.00 (0.0, 0.0)	0.00 (0.0, 0.0)	0.885
Glomerular sclerosis	0.00 (0.0, 1.0)	0.00 (0.0, 1.0)	0.788
Tubular atrophy	1.00 (0.0, 1.8)	1.00 (1.0, 2.0)	0.223
Interstitial fibrosis	1.00 (0.3, 1.8)	1.00 (1.0, 2.0)	0.611
Direct immunofluorescence and electron microscopy			
IgG	2.75 (1.3, 3.0)	2.00 (1.0, 3.0)	0.312
IgA	1.00 (0.6, 1.5)	1.00 (1.0, 2.0)	0.415
IgM	0.50 (0.1, 1.5)	1.00 (1.0, 2.0)	0.134
C3	2.00 (2.0, 2.9)	2.50 (2.0, 3.0)	0.498
C1q	1.00 (1.0, 2.0)	2.00 (1.0, 2.3)	0.155
Fibrin	2.00 (0.0, 2.0)	0.25 (0.0, 1.0)	0.032

*ANCA* Antineutrophil cytoplasmic antibody; *AI* activity index; *CI* chronicity index

On renal pathological evaluation, there was a significantly greater number of total crescents in the ANCA-positive group than in the ANCA-negative group. However, there was no significant difference in the scores for cellular crescents, interstitial inflammation, tubular atrophy or interstitial fibrosis between crescentic LN patients with and without ANCAs.

We did not observe any differences in the activity index (*p* = 0.091) or chronicity index (*p* = 0.980) scores between crescentic LN crescent-positive patients with and without ANCAs. On evaluation of the immunofluorescence parameters, there was no significant difference in the locations of immunoglobulin deposition (IgG, IgA, IgM or C1q) between the two groups, except for the difference in the average intensity of fibrin, which reached statistical significance (*p* = 0.029).

### Treatment regimens and outcomes

There was no significant difference in the treatment algorithm, which included the use of methylprednisolone pulses, oral prednisone, cyclophosphamide, mycophenolate mofetil, plasma exchange or rituximab, between the two groups of ANCA-positive crescentic LN patients and ANCA-negative crescentic LN patients.

The proportion of patients in complete remission and partial remission did not significantly differ between the ANCA-positive group and the ANCA-negative group. During follow-up, ESKD developed in 4/12 (33.33%) patients in the ANCA + group and 14/34 (41.18%) patients in the ANCA- group. The kidney survival rates for ANCA-positive LN patients were 83.3% (10/12) at 1 year, 83.3% (10/12) at 2 years, and 66.7% (8/12) at 3 years. These values were similar to those of the ANCA-negative LN patients, whose survival rates were 79.41% (27/34), 67.6% (23/34), and 58.8% (20/34). During follow-up (average of less than 3 years), there was no significant difference in ESKD or haemodialysis outcomes between the two groups regarding short-term survival (Table 3).

**Table 3:** Treatment responses and follow-up data based on ANCA specificity

	ANCA + (*n* = 12)	ANCA– (*n* = 34)	*p* value
Follow-up time, months	19.50 (6.0, 37.5)	20.00 (9.3, 45.5)	0.842
Treatment			
Methylprednisolone pulse, *n* (%)	11 (91.67)	32 (94.12)	0.77
Oral prednisone, *n* (%)	12 (100)	34 (100)	1.00
Cyclophosphamide i.v, *n* (%)	11 (91.67)	26 (76.47)	0.259
Mycophenolate mofetil, *n* (%)	3 (25.00)	8 (23.53)	0.919
Plasma exchange, *n* (%)	5 (41.67)	11 (32.35)	0.565
Rituximab, *n* (%)	2 (16.67)	1 (2.94)	0.10
Treatment response			
CR, *n* (%)	5 (41.67)	7 (20.59)	0.157
PR, *n* (%)	2 (16.67)	10 (29.41)	0.393
Death, *n* (%)	1 (8.3)	2 (5.9)	0.77
HD, *n* (%)	4 (33.33)	12 (35.29)	0.903
ESKD, *n* (%)	4 (33.33)	14 (41.18)	0.893
SCr, μmol/L at the last follow-up (excluding dialysis patients)	73.75 ± 17.65	100.24 ± 24.62	0.06

*ANCA* Antineutrophil cytoplasmic antibody; *i.v* intravenous; *CR* complete remission; *PR* partial remission; *HD* hemodialysis; *ESKD* end-stage kidney disease

## Discussion

The pathogenesis and manifestations of LN are heterogeneous and variable between and within individuals. Crescent formation is common in LN, especially in patients with a background of proliferative glomerular lesions. It was reported that the percentage of crescents was significantly associated with more severe kidney injury indices, such as the SLEDAI score, serum creatinine concentration, C3 value and several pathologically active indices [10]. Patients with “true” crescentic LN (affecting 50% of the glomeruli) had worse kidney outcomes than did those with pure class IV-G LN, which was also later confirmed by other investigators [11]. The recent development of a more accurate and reproducible histopathological classification system for LN is expected to lead to better disease categorisation. The field is moving towards more personalised treatment approaches.

We found that crescentic glomerulonephritis was not rare in LN, accounting for 8.6% of the total biopsy-proven cases of LN. It was not surprising that patients with crescentic glomerulonephritis had a significantly greater incidence of acute kidney injury and clinical presentation, and worse outcomes. We found that 78.3% of crescentic LN patients presented with acute kidney injury and rapidly progressive glomerulonephritis, and 18/46 (39.1%) crescentic LN patients had developed ESKD at the last follow-up.

It has been reported that ANCA positivity rates range from 0 to 30% in LN [2, 4]. The role of ANCAs is unclear and debated [12]. Although most authors have indicated that ANCA seropositivity is common in patients with SLE, especially in those with disproportionate necrotising and crescentic features, several studies have failed to find a correlation between ANCAs and LN [13, 14]. The exact role of ANCAs in the pathologic course of crescentic LN has not been determined. In our study, ANCA positivity accounted for 18.8% of the total number of biopsy-proven crescentic LN cases. To investigate this phenomenon, we compared the clinical and pathological characteristics of patients who were and were not ANCA positive. We observed a significantly lower level of proteinuria, a lower proportion of nephrotic syndrome (*p* < 0.05) and a higher level of serum albumin (*p* < 0.05) in the ANCA-positive group than in the ANCA-negative group. Recently, several studies have shown that ANCA positivity may correlate with more haematuria, more proteinuria and higher creatinine levels [15, 16]. Other studies suggest that ANCA-positivity has no correlation with clinical indicators. We found that the urine protein level of ANCA-positive patients was lower than that of ANCA-negative patients and that the number of patients with nephrotic syndrome was lower. Moreover, the rate of acute kidney injury was not greater in ANCA-positive patients than in ANCA-negative patients. However, whether ANCA positivity is associated with SLEDAI score has not been determined [17]. We did not observe any differences in the extrarenal manifestations, C3 levels, SLEDAI values or serum ANA antibody positivity between crescentic LN patients with and without ANCAs. These results were not in accordance with most of the previously reported ones [2].

Positive ANCA antibody serology in patients with LN has been associated with distinct histopathological features on renal biopsy. It has been reported that patients with LN and positive ANCA serology are more likely to have segmental endocapillary hypercellularity and cellular fibroid crescents on renal biopsy (ISN/RPS Class III, IV-S, IV-G LN) [2, 4, 14] than are ANCA-negative patients. In our study, the total percentage of crescents was greater in the ANCA-positive group than in the ANCA-negative group; however, this difference in the proportion of total crescents in the glomeruli was not statistically significant. Moreover, there were no significant differences in the scores for cellular crescents, interstitial inflammation, tubular atrophy or interstitial fibrosis between the ANCA-positive and ANCA-negative groups.

It has been suggested that LN might facilitate the process of ANCA formation by promoting neutrophil degranulation and priming neutrophils, thus increasing the surface expression of ANCAs. However, the effect of ANCAs on the prognosis of patients with crescentic LN and on the remission rate has not been studied so far [18]. In our retrospective study, although the ANCA-negative group had a slightly greater incidence of acute kidney disease, there was no significant difference between the two groups. In accordance with our results, a study reported that there were no significant differences in therapy between LN patients with and without ANCAs [5]. Several recent studies have reported worse kidney survival in ANCA-associated vasculitis (AAV) patients. ANCA positivity is most likely related to smouldering disease, which causes ESKD [19]. Currently, there are rare reports on ANCAs and crescentic lupus nephritis. Abe et al. reported a patient with systemic lupus erythematosus who developed macrohematuria and massive proteinuria with pathological formation of fibrous crescents after seroconversion of MPO-ANCA, and suggested that tacrolimus might be a useful immunosuppressant for treating patients with progressive LN with MPO-ANCA [20]. We have summarised the clinical and pathological characteristics and clinical decision-making tendencies of previous ANCA and lupus nephritis in Table 4. Some studies have shown that rituximab was administered more frequently in ANCA-positive LN patients [21]. It was also reported that cyclophosphamide and mycophenolate mofetil might be useful for patients with progressive LN and MPO-ANCAs [2, 21]. Most studies have shown that there were no significant differences in therapy between LN patients with and without ANCAs [6, 20]. Further study of lupus pathology and ANCA subtype classification may be meaningful for the treatment of patients. However, we could not draw this conclusion from our study, and we observed no association between ANCAs and kidney outcomes in crescentic patients. There was no statistically significant difference in the initial treatment of immunosuppressants between the two groups. ESKD developed in 4/12 (33.33%) ANCA-positive LN patients and in 14/34 (41.18%) ANCA-negative LN patients in our study, but the difference was not significant. We showed that the percentage of ANCA-positivity was quite high in patients with crescentic lupus, possibly because of the formation of crescent-shaped LN. However, unlike what we assumed, based on our data, ANCA positivity did not seem to be related to disease activity, histopathologic features or kidney outcomes.

**Table 4:** The clinical, pathological relationship and treatment tendency between ANCA and lupus nephritis

Trial	Time/country	Design	*N* (ANCA + vs ANCA-)	ANCA + prevalence	Clinical relation with ANCA +	Histopathologic features relation with ANCA +	Treatment difference with ANCA +	The response to treatment with ANCA +
Wang ying.et al. [22]	2023/China	Observational cohort, retrospective	46 vs. 442	22.4%	ALB↑ Crea↓ Anti-Sm↑ ANA↑	Class III↓ Class IV↓ Focal endocapillary hypercellularity↓ tubulointerstitial fibrosis↑	ND	PR + CR↓
Rosanna Lacetera et al. [21]	2023/Italy	Retrospective multicentre study	16 vs. 100	17.9%	Acute nephritic syndrome↑ Proliferative histological class↑↑	Class III + IV↑ Class IV↑ Activity index↑	Rituximab↑ Cyc↑	NO
Ying Pan et al. [23]	2021/China	Observational cohort, retrospective	45 vs. 75	37.5%	Anti-dsDNA↑ SLEDAI↑	NO	ND	ND
Dina Said et al. [19]	2021/Egypt	Cross-sectional study	16 vs. 44	16.84%	SLEDAI↑ C3↓ Crea↑	Mesangio proliferative LN↑ Diffuse proliferative LN↑	NO	ND
Wang et al. [6]	2020/China	Observational cohort, retrospective	36 vs. 247	12.7%	Anti-dsDNA↑ Crea↑	Diffuse proliferative LN↑	NO	Kidney survival rate↓
Shuai Wang et al. [6]	2020/China	Observational cohort, retrospective	36 vs. 247	12.7%	Anti-dsDNA↑ Crea↑ C3↓	NO	NO	CR + PR↑ Mortality rate due to pulmonary infection↑
Pyo.et al. [24]	2019/China	Observational cohort, retrospective	12 vs. 79	13.2%	ALB↓ Anti-dsDNA↑ SLEDAI↑	Chronicity indices↑	ND	NO
Tabitha Turner-Stokes et al. [14]	2017/UK	Observational cohort, retrospective	32 vs. 222	12.6%	Anti-dsDNA↑ C4↓ Crea↑	Class IV-S↑	Cyc↑	NO
Wang et al. [4]	2016/China	Observational cohort, retrospective	26 vs. 128	16.8%	Oral ulcerations↑ Alopecia↑ C3↓ Anti-dsDNA↑	Glomerular sclerosis↑ Chronic index↑	Cyc↑ MMF↑	PR + CR↑ Kidney survival rate↓
Abe et al. [20]	2015/Japan	Case report	One case	One case	C3↓ C4↓	Class III A/C) with a fibrous crescent formation	Tacrolimus was recommended	CR
Su et al. [25]	2015/China	Cross-sectional descriptive analytical study	45 vs. 62	42%	Raynaud’s Phenomenon↑ Interstitial lung disease↑ C3↓ C4↓	ND	ND	ND
Nishiya et al. [1]	1997/Japan	Observational cohort, retrospective	13 vs. 18	42%	SLEDAI↑	NO	ND	ND

*ANCA* Antineutrophil cytoplasmic antibody; *N* number of patients; *ND* not documented; *NO* no significant difference; *ALB* serum albumin; *Scr* serum creatinine; *C3* serum C3; *C4* serum C4; *ANA* antinuclear antibodies; *ds-DNA* double-stranded deoxyribonucleic acid; *Anti-Sm* anti-Smith antibody; *Cyc* cyclophosphamide; *Tac* tacrolimus; *MMF* mycophenolate mofetil; *SLEDAI* systemic lupus erythematosus disease activity index; *CR* complete remission; *PR* partial remisson

The following factors may have contributed to our results. First, our data were obtained from lupus patients at three medical centres, and the sample size was small. Second, the detection method used for ANCAs may be nonspecific. In addition, ANCAs may not be the only antibodies involved, and some new markers are still under study. Third, the mean follow-up time for all our patients was 20 (6.75, 45) months. Most patients were followed up for less than 3 years, which may have affected the final results. Furthermore, all patients included in this study were of Chinese Han ethnicity.

In conclusion, ANCAs may be involved in the pathological changes observed in crescentic lupus nephropathy. However, ANCA positivity was not related to disease activity, histopathological features or short-term kidney outcomes. Within its limits, our study suggests that crescentic lupus nephritis with ANCA may not require a more aggressive treatment. However, further studies are needed to confirm this hypothesis.

## Data Availability

The data that support the findings of this study are available from the corresponding author, upon reasonable request.
